# Trends in suicides among italian youth aged 10 to 25: A nationwide register study

**DOI:** 10.1192/j.eurpsy.2021.461

**Published:** 2021-08-13

**Authors:** A. Forte, M. Vichi, S. Ghirini, M. Orri, M. Pompili

**Affiliations:** 1 Suicide Prevention Center, Sapienza University of Rome, Italy, Rome, Italy; 2 Department Of Neurosciences, Mental Health And Sensory Organs, Suicide Prevention Center, Sant’andrea Hospital, Sapienza University Of Rome, Rome, Italy, Sapienza university of Rome, ROMA, Italy; 3 Statistical Service, Istituto Superiore di Sanità, National Institute of Health (ISS), Rome, Italy; 4 Department Of Psychiatry, McGill Group for Suicide Studies, Douglas Mental Health University Institute, McGill University, Montreal, Canada, Montreal, Canada; 5 Department Of Neurosciences, Mental Health And Sensory Organs, Suicide Prevention Center, Sant’andrea Hospital, Sapienza University Of Rome, Rome, Italy, Sapienza university of Rome, roma, Italy

**Keywords:** adolescence, Suicide

## Abstract

**Introduction:**

Suicide continues to be a significant cause of mortality in most countries worldwide, especially among youth. Documenting current trends and sources of variation in youth suicide rates is critical to inform prevention strategies.

**Objectives:**

We aimed to 1. document suicide mortality trends among Italian youth from 1981 to 2016 2. describe age, sex, and urbanization specific suicide rates in this period, and 3. describe suicide methods and their change over time.

**Methods:**

We relyed on official mortality data for the period 1981-2016 for adolescents and young adults (ages 10-25 years). We estimated standardized all-cause and suicide mortality rates per 100,000 individuals and used Joinpoint regression analysis to determine annual mortality trends and statistically significant changes in rate trends. Analyses were reported by sex, age group, urbanization level and suicide method.

**Results:**

From 1981 to 2016, 1,752 suicides were identified among youth aged 10-17 (boys/girls ratio in 2016, 5.3) and 9,897 among youth aged 18-25 years (boys/girls ratio in 2016, 4.0). While the all-cause mortality rate decreased over time for both boys and girls, overall suicide rates remained stable for boys and showed a small decrease for girls. For boys, suicide was most common in rural than to metropolitan areas, while it was the opposite for girls. The most common method for boys was hanging, while for girls was fall.

**Conclusions:**

Differently from other countries, youth suicides were stable (boys) or slightly declining (girls). We found differences according to the urban vs. rural areas. Factors influencing these trends and sex differences are crucial in delivering prevention strategies.

**Disclosure:**

No significant relationships.

